# The role of Toll-like receptor 4 in apoptosis of brain tissue after induction of intracerebral hemorrhage

**DOI:** 10.1186/s12974-019-1634-x

**Published:** 2019-11-26

**Authors:** Xiaowei Fei, Yeting He, Jia Chen, Weitao Man, Chen Chen, Kai Sun, Boyun Ding, Chongwu Wang, Ruxiang Xu

**Affiliations:** 1 Department of Neurosurgery, Sichuan Academy of Medical Sciences and Sichuan Provincial People’s Hospital, School of Medicine, University of Electronic Science and Technology of China, Chengdu, 610072 Sichuan China; 2 Affiliated Bayi Brain Hospital, General Army Hospital, Beijing, 10000 China; 3 Dapartment of Physiology, Dalian Medical University, Dalian, 116044 China; 4 Department of Neurosurgery, Second Affiliated Hospital of Dalian Medical University, Dalian, 116044 China; 5 Affiliated BaYi Children’s Hospital, Clinical Medical College in The Seventh Medical Center of PLA General Hospital, Southern Medical University, Beijing, China; 6 Department of Neurosurgery, Beijing Tsinghua Changgung Hospital, School of Clinical Medicine, Tsinghua University, Beijing, 102218 China

**Keywords:** Toll-like receptor 4, Apoptosis, Intracerebral hemorrhage, TNF-α, IL-1β

## Abstract

**Background:**

Inflammation and apoptosis caused by intracerebral hemorrhage (ICH) are two important factors that affect patient prognosis and survival. Toll-like receptor 4 (TLR4) triggers activation of the inflammatory pathway, causing synthesis and release of inflammatory factors. The inflammatory environment also causes neuronal apoptosis. However, no studies have reported the role of TLR4 in inflammation and apoptosis.

**Methods:**

We performed survival curve analysis and behavioral scores on TLR4 knockout mice and wild-type mice after inducing ICH. We used TLR4 knockout mice and wild-type mice to make ICH models with type VII collagenase and explored the link between TLR4 in inflammation and apoptosis. We used Western blot to detect the expression of apoptosis-related proteins, inflammatory factors, and their receptors at different time points after ICH induction. The effects of TLR4 on apoptosis were observed by TUNEL, Hoechst, and HE staining techniques. The association with TLR4 in inflammation and apoptosis was explored using IL-1β and TNF-α antagonists. Data conforming to a normal distribution are expressed as mean ± standard deviation. Grade and quantitative data were compared with rank sum test and *t* test between two groups. *P* < 0.05 was considered statistically significant.

**Results:**

TLR4 knockout significantly increased the survival rate of ICH mice. The scores of TLR4 knockout mice were significantly lower than those of wild-type mice. We found that TLR4 knockout mice significantly inhibited apoptosis and the expression of inflammatory factors after the induction of ICH. The apoptosis of ICH-induced mice was significantly improved after injecting IL-1β and TNF-α antagonists. Moreover, the anti-apoptotic effect of the antagonist in wild-type mice is more pronounced. A single injection of the antagonist failed to improve apoptosis in TLR4 knockout mice.

**Conclusions:**

We conclude that TLR4-induced inflammation after ICH promotes neuronal apoptosis. IL-1β and TNF-α antagonists attenuate this apoptotic effect. Therefore, targeting TLR4 in patients with clinical ICH may attenuate inflammatory response, thereby attenuating apoptosis and improving prognosis.

## Introduction

Intracerebral hemorrhage (ICH) refers to the rupture of blood vessels in the brain that causes bleeding. When the blood vessels are destroyed, the blood flowing out presses the surrounding brain tissue to cause damage to the neurons [1]. Hypertension with small arteriosclerosis, microaneurysm, or microangioma is a common cause of cerebral hemorrhage [2]. Inflammatory damage is an important factor contributing to brain damage after ICH. The initial process is the activation of microglia, followed by inflammatory cell exudation, release of inflammatory mediators, and cross-response [3]. Pathological changes in brain tissue such as blood-brain barrier destruction [4], tissue edema, and cell death are caused by a variety of different signaling pathways [5]. In recent years, researches have been done on the mechanism of apoptosis caused by inflammatory injury after ICH. Various inflammatory injury signaling pathways have been suggested as possible targeted therapy of ICH, but the clinical mortality and disability rates have not improved significantly.

Members of the Toll-like receptor (TLR) family are closely related to a series of TLRs in *Drosophila* and play an important role in the innate immune response [6–9]. TLR recognizes conserved motifs in various pathogens and mediates defense responses [10–12]. Triggering the TLR pathway often leads to activation of nuclear factor κB (NF-κB) and subsequent regulation of immune and inflammatory genes [9]. Members of the TLR and IL-1 receptor families share a conserved region of approximately 200 amino acids [8]. TLR4 recognizes and initiates the lipopolysaccharide (LPS)-triggered immune response of gram-negative bacteria [11]. TLR4 triggers activation of the NF-κB, interferon regulatory factor 3 (IRF-3), and mitogen-activated protein kinase (MAPK) pathways, causing inflammatory cytokine synthesis [13].

Many studies have reported the role of TLR4 in ICH. Liu et. al. (2016) reported that peroxiredoxin-1-mediated activation of the TLR4/NF-κB pathway contributes to neuroinflammatory damage in ICH [14]. Fang et al. (2014) found that CD36 mediates hematoma absorption through negative regulation of TLR4 signaling after cerebral hemorrhage [15]. Lin et al. (2012) suggested that Heme activates TLR4-mediated inflammatory injury via MyD88/TRIF signaling pathway in intracerebral hemorrhage [16]. Although many researchers have reported the role of TLR4 in ICH, current studies on the relationship between TLR4 and apoptosis are often mixed with other variables, and no specific studies have been conducted for identifying the specific relationship between TLR4 and apoptosis after ICH induction. Therefore, we used TLR4 knockout mice to explore the role and underlying mechanism of TLR4 in brain tissue apoptosis after ICH induction.

## Materials and methods

### Polymerase chain reaction

Mouse tails were cut and digested with proteinase K for 20 min at 55 °C, and further inactivated with protein K for 5 min at 100 °C. Polymerase chain reaction (PCR) was performed according to the protocol in One Step Mouse Genotyping Kit (Vazyme; China). The primers for TLR4-Mut were (forward) 5′-GCA AGT TTC TAT ATG CAT TCT C-3′ and (reverse) 5′-CCT CCA TTT CCA ATA GGT AG-3′. The primers for TLR4-Wt were (forward) 5′-ATA TGC ATG ATC AAC ACC ACA G-3′ and (reverse) 5′-TTT CCA TTG CTG CCC TAT AG-3′. PCR results were detected by agarose gel electrophoresis.

### Reverse transcription-polymerase chain reaction

We harvested cells and extracted RNA from the cells using the TRIzol method. Reverse transcription was performed according to the protocol in the HiScript II Q Select RT SuperMix for qPCR (+gDNA wiper) kit (Vazyme; China). qPCR was performed according to the protocol in the ChamQ SYBR Color qPCR Master Mix (Low ROX Premixed) kit (Vazyme; China). TLR4 primers were designed by Wcgene Biotech Co., Ltd., Shanghai, China and purchased from Sangon Biotech Co., Ltd., Shanghai, China. The primers for TLR4 were (forward) 5′-TGT TCC TTT CCT GCC TGA GAC-3′ and (reverse) 5′-GGT TCT TGG TTG AAT AAG GGA TGT C-3′. The expression of related RNA was calculated by the 2−ΔΔCt method, and GAPDH was used as a control. The experiment was repeated three times.

### ICH model

All animal experiments were conducted according to protocols approved by the Institutional Ethics Committee of the Seventh Medical Center of the Chinese People's Liberation Army General Hospital. C57 and TLR4-knock out C57 mice (200 each) were purchased from Shenzhen Huafukang Bioscience Co., Inc., (Shenzhen, China) and Nanjing Biomedical Research Institute of Nanjing University, Nanjing, China, respectively. Mice were anesthetized with 4% chloral hydrate (400 mg/kg) injected intraperitoneally. Rectal temperature was maintained at 37.5 °C. Stereotactic technique was used to make a scalp incision along the midline and drill a burr hole on the left side of the skull (2 mm posterior and 1 mm lateral of the bregma). One microliter collagenase VII (0.5 U/μl) (Sigma; USA) was transferred into a 1 μl Hamilton syringe. The syringe was connected to a microinjection pump, needle inserted into the brain through the burr hole (depth, 2.8 mm from the bone surface), and 0.2 μl collagenase was injected within 10 min. The syringe was withdrawn after 10 min and the inhibitors IL-1A and R7050 (3 mg/kg, dissolved in normal saline) were reinjected in situ in 5 min. After the surgery, the skull hole was sealed with bone wax, incision was closed with sutures, and mice were allowed to recover. To avoid postsurgical dehydration, 0.5 ml normal saline was subcutaneously injected to each mouse immediately after surgery. ICH model mice were sacrificed at different time points for staining and Western blot.

### Experimental grouping

Immunofluorescence experiments used ten TLR4(+/+)(control) and ten TLR4(+/+) (ICH) mice.

Survival curve analysis and behavioral score calculation was done on ten TLR4+/+ mice and ten TLR4−/− mice.

Western blot used 40 TLR4+/+ and 40 TLR4−/− mice, with ten mice in each group being sacrificed on days 1, 3, and 7 for detection of apoptotic and inflammatory proteins. The remaining ten mice in each group did not make an ICH model for comparison. The Western blot was repeated thrice for each sample.

A total of 30 TLR4+/+ and 30 TLR4−/− mice (ten mice in each group) were sacrificed on days 1, 3, and 7 for staining experiments to detect apoptosis. TUNEL stained samples (*n* = 10 in each group) and Hoechst stained samples (*n* = 40 in each group) were observed under low and high magnifications, respectively. Four fields were randomly selected in each Hoechst stained sample and the cells were counted. The edema area of each HE stained sample (*n* = 10 in each group) was calculated.

A total of 80 TLR4+/+ and 80 TLR4−/− mice (five in each group) were sacrificed for TUNEL and Hoechst staining to detect apoptosis in brain tissue after injecting antagonists of inflammatory factors. TUNEL stained samples (*n* = 5 in each group) and Hoechst stained samples (*n* = 20 in each group) were observed under low and high magnifications, respectively. Four fields were randomly selected in each Hoechst stained sample and the cells were counted.

After injecting antagonists of inflammatory factors, survival curve analysis and behavioral score calculation was done on 20 TLR4+/+ and 20 TLR4−/− mice (ten mice in each group).

### Brain tissue frozen section selection

All experiments on staining including HE, TUNEL, and Hoechst staining and immunofluorescence were performed on frozen sections of brain tissue. The brain tissue was fixed with 4% paraformaldehyde and subjected to gradient dehydration with a sucrose solution, followed by frozen sectioning on the coronal plane. Five sections with the largest bleeding area were selected for subsequent experiments.

### Co-immunoprecipitation

An appropriate amount of brain tissue was taken from the bleeding site on the third day after inducing ICH and 3 ml pre-cooled RIPA lysate was added. The tissue was ground using a tissue homogenizer and placed on ice for 30 min. Tissue debris was removed by centrifugation at 10,000×*g* for 10 min in 4 °C. The supernatant was collected in a 15 ml centrifuge tube. In a 1.5 ml microcentrifuge tube, 1 ml supernatant was mixed with 0.2 μg primary antibody and incubated at 4 °C for 1 h. The solution was incubated overnight with 20 μl protein A/G Plus-agarose (Santa Cruz; China) on a rotary mixer at 4 °C. The beads were collected by centrifugation at 2500 rpm for 5 min in 4 °C and washed thrice with PBS. After washing the beads, 40 μl 1X electrophoresis loading buffer was added and boiled in a 100 °C water bath for 10 min. After centrifugation at 2500 rpm for 5 min in 4 °C, the supernatant was collected and used for Western blot. The following primary antibodies were used: rat anti-TLR4 (1:500; Abcam) for IP; rat anti-TLR4 (1:1000; Abcam); rabbit anti-CD14 (1:1000; Abcam); and rabbit anti-collagenase (1:10,000; Abcam) for Western blot.

### Bimolecular fluorescence complementation

We selected the yellow fluorescent protein sequence in the addgene database, and the plasmid sequence was designed by Genechem Co., Ltd., Shanghai, China and purchased from Genechem Co., Ltd., Shanghai, China. After amplification of the glycerol bacteria, the plasmid was extracted using a plasmid extraction kit (Cwbiotech, China). The extracted plasmid was transferred into BV2 cells using jetPRIME DNA transfection reagent (Polyplus, France), and DMEM media (Gibco, USA) supplemented with 10% serum (Gibco, USA) was replaced in 12 h. BV2 cells were incubated at 37 °C with 5% CO_2_ for 48 h and were observed at a wavelength of 515 nm using a fluorescence microscope. The experimental procedure was carried out in strict accordance with the kit instructions.

### Western blot

Mice were anesthetized at different time points after inducing ICH and the brain tissue around the bleeding site was used for Western blot. Protein extraction solution (200 μl) (Sigma; USA) was added, then the brain tissue was ground with a tissue homogenizer, lysed on ice for 30 min, and incubated with 50 μl 5X SDS-PAGE loading buffer for 10 min. Samples were electrophoretically separated on a 12% SDS-PAGE gel (30 mg total protein/lane). After transfer to PVDF membrane, proteins were blocked with 5% BSA (Sigma; USA) for 1 h and incubated overnight at 4 °C with the primary antibody. The following primary antibodies were used: rabbit anti-TNF-α (1:1000; Abcam), rabbit anti-IL-1β (1:2500; Abcam), rabbit anti-IL-1 Receptor I (1:1000; Abcam), rabbit anti-TNF Receptor II (1:10,000; Abcam), rabbit anti-Bax (1:1000; Abcam), rabbit anti-Bcl-2 (1:2000; Abcam), rabbit anti-Caspase 8 (1:1000; Abcam), rabbit anti-Caspase 9 (1:2000; Abcam), rabbit anti-TLR4 (1:500; Abcam), rabbit anti-MyD88 (1:1000; Abcam), rabbit anti-IKB alpha (phospho S36) (1:10,000; Abcam), rabbit anti-IKK alpha (1:10,000; Abcam), rabbit anti-NF-κB p65 antibody (1:2000; Abcam), rabbit anti-NF-kB p65 (phospho S276) (1:1000; Abcam), rabbit anti-Phospho-NF-κB p65 (Ser536) (1:1000; CST), and rabbit anti-Caspase 3 (1:1000; CST). Membranes were washed thrice with TBST and incubated with goat anti-mouse/rabbit secondary antibody (1:10,000; Abcam) for 2 h at 25–27 °C. After TBST wash, membranes were scanned by ECL and analyzed by gel imaging system (Bio-Rad).

### Hoechst staining

Frozen sections of the brain tissue were washed twice with PBS to remove the tissue embedding agent (OCT) and stained with 0.5 ml Hoechst staining solution (Beyotime; China) for 5 min with manual shaking several times. The staining solution was removed, sections washed twice with PBS for 3 min each time, and the liquid was drained. The section was carefully placed on a slide with a drop of anti-quenching seal and covered with a clean cover slip to avoid air bubbles. A blue nucleus was detected by a fluorescence microscope. The excitation and emission wavelengths were about 350 and 460 nm, respectively.

### TUNEL staining

Frozen sections were washed twice with PBS for 3 min each time and incubated in PBS containing 0.5% Triton™ X-100 for 5 min at room temperature. The incubated tissue sections were washed twice with PBS for 3 min each time. The sections were further incubated with 50 μl TUNEL test solution (Beyotime; China) at 37 °C for 1 h in dark. After incubation, they were washed with PBS three times for 3 min each time and observed under a fluorescence microscope. The excitation and emission wavelengths were 550 and 570 nm, respectively.

### HE staining

The frozen sections of brain tissue were placed in an oven at 37 °C for 10 min to dissolve the tissue embedding agent and washed. The staining process was carried out as follows: Hematoxylin for 3 min, water wash, hydrochloric ethanol for 10 s, water wash, 1% ammonia for 10 s, water wash, eosin for 1 min and 30 s, water wash, 75% alcohol for 10 se, anhydrous alcohol for 10 s, xylene for 2 s, neutral resin mount, and observation under a microscope.

### Immunofluorescence

Brain tissue was fixed with 4% paraformaldehyde for 48 h, dehydrated with 15% and 30% sucrose, and then frozen and sectioned. Sections were blocked with blocking buffer (1× PBS/5% BSA/0.3% Triton™ X-100) for 1 h at room temperature. The primary antibody was diluted in a dilution buffer (1× PBS/1% BSA/0.3% Triton™ X-100). The following primary antibodies were used: rabbit anti-NeuN (1:300; Abcam), goat anti-GFAP (1:300; Abcam), and rabbit anti-Iba1 (1:100; Abcam). Tissue sections were incubated with primary antibody overnight at 4 °C, washed thrice with PBS, and incubated with fluorescent secondary antibody for 1 h at room temperature in dark. They were then washed three times with PBS, mounted with DAPI, and observed under a confocal microscope.

### Statistical analysis

All experiments were repeated three times and SPSS 17.0 software was used for data statistics. Measurement data that conforms to a normal distribution are expressed as mean ± standard deviation. Grade and quantitative data were compared with rank sum test and *t* test between two groups. *P* < 0.05 was considered statistically significant.

## Results

### Collagenase does not interact with TLR4 and TLR4 knockout improves symptoms and survival rate in mice after ICH induction

**Fig. 1:**
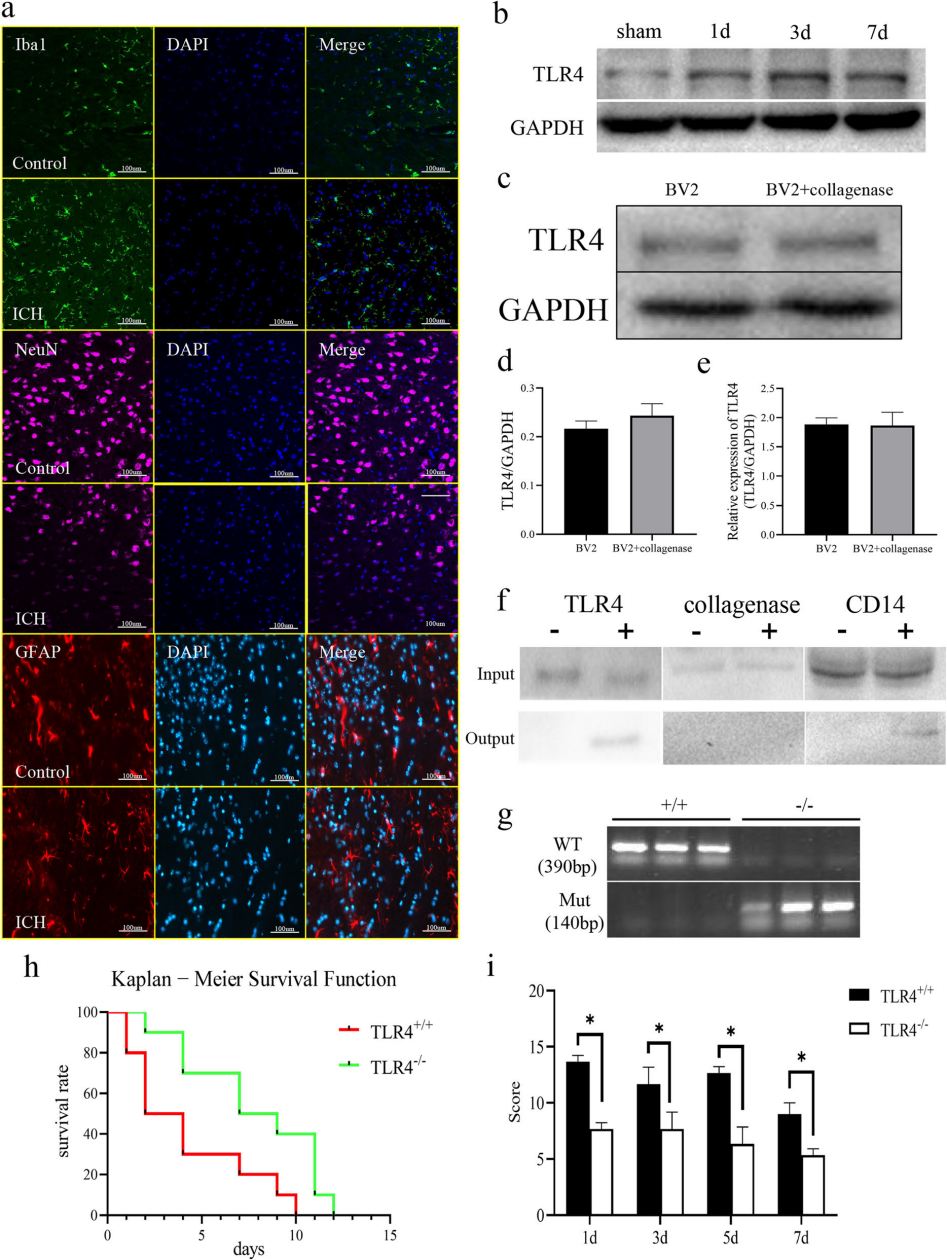
TLR4 knockout increases survival and reduces behavioral scores in ICH-induced mice. **a** Immunofluorescence staining of brain tissue for IBA-1 (488 nm), NeuN (647 nm), and GFAP (555 nm) in hemorrhagic site. **b** Changes in TLR4 expression at the first, third, and seventh day after inducing ICH. **c** Detection of TLR4 expression in BV2 cells treated with collagenase VII for 48 hours. **d** Quantification of TLR4 expression in panel **c**. **e** Transcriptional level of TLR4. **f** Interaction of TLR4 with collagenase VII as detected by immunoprecipitation ; positive control CD14. **g** Genetic identification of wild-type and knockout mice using mutant TLR4 primers and wild-type TLR4 primers. **h** Kaplan-Meier survival analysis of 10 TLR4+/+ mice and 10 TLR4−/− mice. **i** Behavioral Longa scores of ICH-induced mice on postoperative days 1, 3, 5, and 7. The difference was statistically significant. *P < 0.05 represents a statistically significant difference between the two groups

**Fig. 2:**
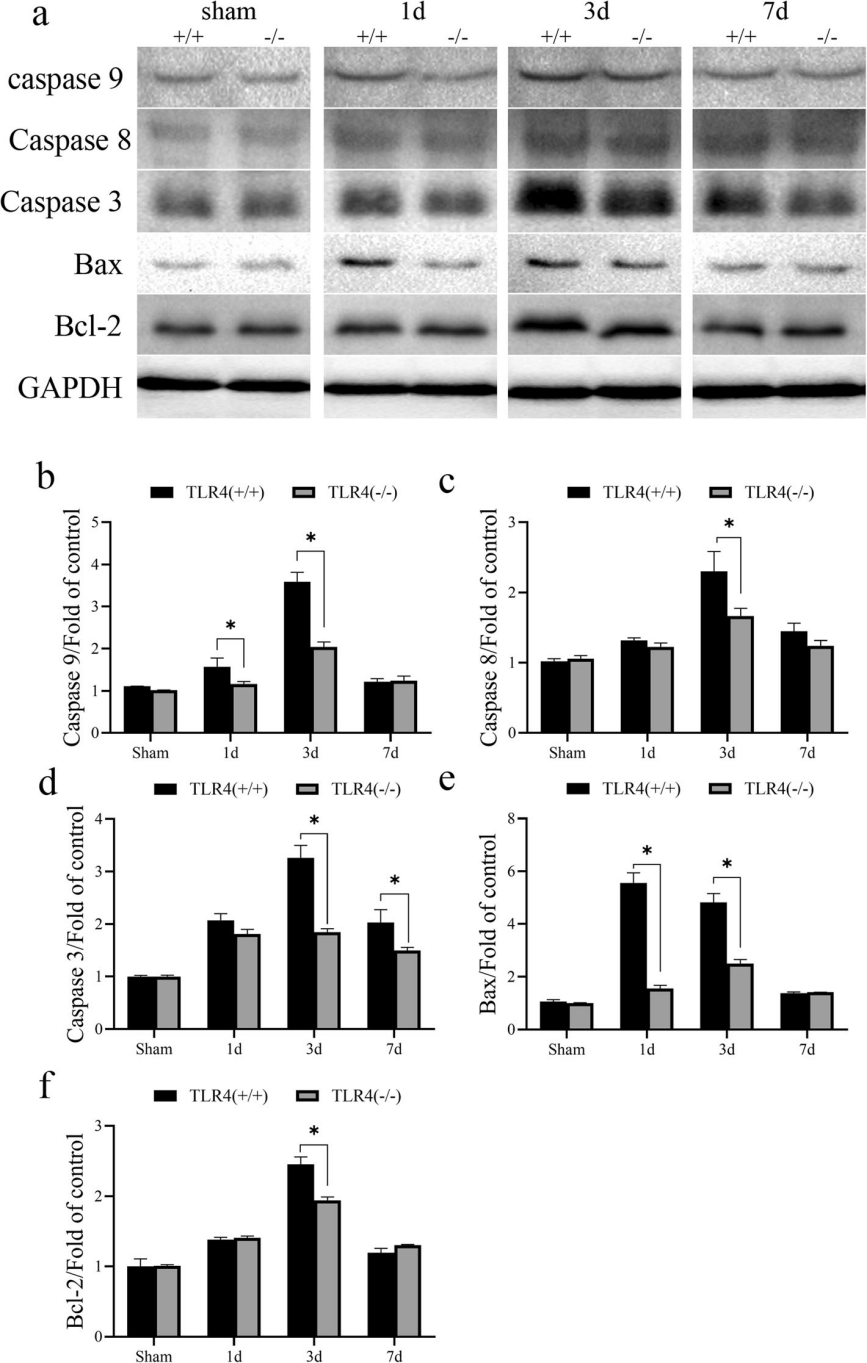
Differential expression of apoptosis-related proteins in TLR4 knockout mice and wild-type mice after ICH induction. **a** The expression of Caspase 3, 8, 9, Bcl-1 and Bax in the brain tissue around the bleeding site of TLR4 knockout mice and wild-type mice on days 1, 3, and 7 after ICH induction. **b** Quantification of caspase 9. **c** Quantification of caspase 8. **d** Quantification of caspase 3. **e** Quantification of Bax. **f** Quantification of Bcl-2. Western total protein was 30 μg. GAPDH is used as a housekeeping protein to prove the equal loading in each lane of the electrophoresis and normalize densitometric values of the other protein analyzed. Each experiment was repeated three times. *P < 0.05 represents a statistically significant difference between the two groups

First, we induced ICH in wild-type mice and performed immunofluorescence staining for IBa-1, NeuN, and GFAP, specific markers for microglia, neurons, and astrocytes, respectively, on the bleeding site on the third day after surgery. We found that ICH caused activation of microglia and neuronal apoptosis in the brain tissue surrounding the bleeding site, but had no significant effect on astrocytes (Fig. 1a). We measured the expression of TLR4 in the brain tissue at the bleeding site on the first, third, and seventh postoperative days and found that TLR4 expression increased significantly and reached its highest level on the third day. Although the expression of TLR4 showed a downward trend on the seventh day, it was still higher than that in the sham group (Fig. 2b). To verify whether collagenase interacts with TLR4, we applied collagenase (1 U/ml) to mouse microglia BV2 for 48 h in vitro and found that collagenase treatment did not affect transcription and translation levels of TLR4 (Fig. 1c–e). In addition, in order to verify the results of in vitro cell experiments, we performed an immunoprecipitation experiment using CD14 as a positive control [17] in vivo to explore whether TLR4 interacts with collagenase. Immunoprecipitation showed that TLR4 interacted with the positive control CD14 but not with collagenase (Fig. 1f). In addition, we designed the bimolecular fluorescence complementation (BiFC0 experiment, selected the yellow fluorescent protein sequence in addgene, designed the plasmid, and transfected into BV2 cells. We did not observe the appearance of fluorescence. Therefore, combined with in vivo co-immunoprecipitation (Co-IP) experiments, we believe that TLR4 and collagenase have no interaction.

We identified TLR4 knockout mice by PCR (Fig. 1g). After successfully developing the mouse ICH model, we analyzed the survival rate of mice by Kaplan-Meier method and found that TLR4 knockout significantly increased the survival rate of ICH-induced mice (Mantel-Cox test, Chi-square 5.695, *P* = 0.017) (Fig. 1h). We calculated behavioral Longa scores at multiple time points and found that the scores of TLR4 knockout mice were significantly lower than those of wild-type mice (Fig. 1i). The results are statistically significant. In addition, we found that postoperative mice developed significant behavioral symptoms and gradually reduced symptoms over a long period of time.

### TLR4 knockout inhibits the expression of apoptosis-related proteins after ICH induction

To examine the effect of TLR4 on apoptosis in brain tissue after ICH induction, we examined the expression of apoptotic proteins caspase 3, 8, 9, Bcl-1, and Bax in the brain tissue around the bleeding site on days 1, 3, and 7 after inducing ICH. The caspase family and the bcl/bax family of proteins are classical apoptosis pathway regulatory proteins [18–20]. We found that compared with the expression in the brain tissue of control group mice, the expression of apoptotic proteins in the brain tissue of ICH-induced mice increased significantly in a time-dependent manner. On the first day after surgery, caspase 9 and Bax were expressed in TLR4(−/−) at a lower level than in the wild-type mice and the difference was statistically significant (Fig. 2b,e). On postoperative day 3, all detected apoptotic proteins were less expressed in TLR4(−/−) than in the wild-type mice with the difference being statistically significant (Fig. 2b–f). On postoperative day 7, due to the ability of the mice to self-recover, only the expression of caspase 3 was significantly different between the two groups (Fig. 2d).

### TLR4 knockout inhibits apoptosis of brain cells after ICH induction

**Fig. 3:**
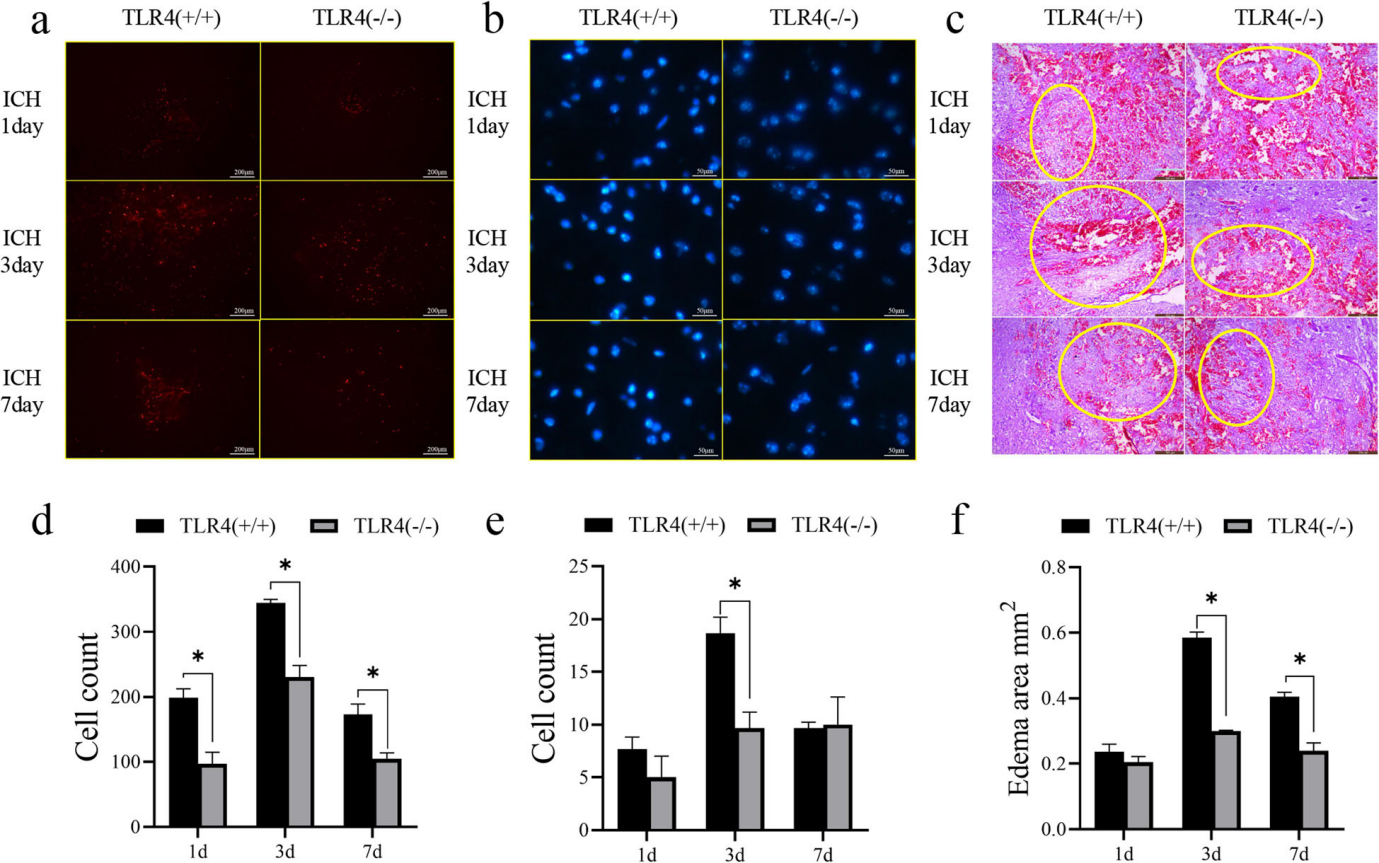
Comparison of apoptosis in brain tissue of TLR4 knockout mice and wild-type mice after inducing ICH. **a** TUNEL staining, **b** Hoechst staining, and **c** HE staining of brain tissue of TLR4 knockout mice and wild-type mice on days 1, 3, and 7 after ICH induction. **d** Quantification of TUNEL staining. **e** Quantification of Hoechst staining. **f** Quantification of HE staining. *P < 0.05 represents a statistically significant difference between the two groups

To further examine the effects of TLR4 on brain cell apoptosis after ICH induction, we performed TUNEL (Fig. 3a) and Hoechst (Fig. 3b) staining techniques on brain tissue on days 1, 3, and 7 after surgery and found that TLR4 knockout mice had fewer dead cells at the site of cerebral hemorrhage than the wild-type mice. In addition, HE staining showed that TLR4 knockout mice exhibited lighter brain edema and clearer cell contours after the induction of ICH than the wild-type mice (Fig. 3c). The staining results were quantified and the differences were found to be statistically significant (Fig. 3d–f). Changes in apoptosis on the timeline of the two groups of mice were consistent with changes in expression of apoptotic proteins (Fig. 2).

### TLR4 knockout inhibits ICH-induced inflammatory factor release

**Fig. 4:**
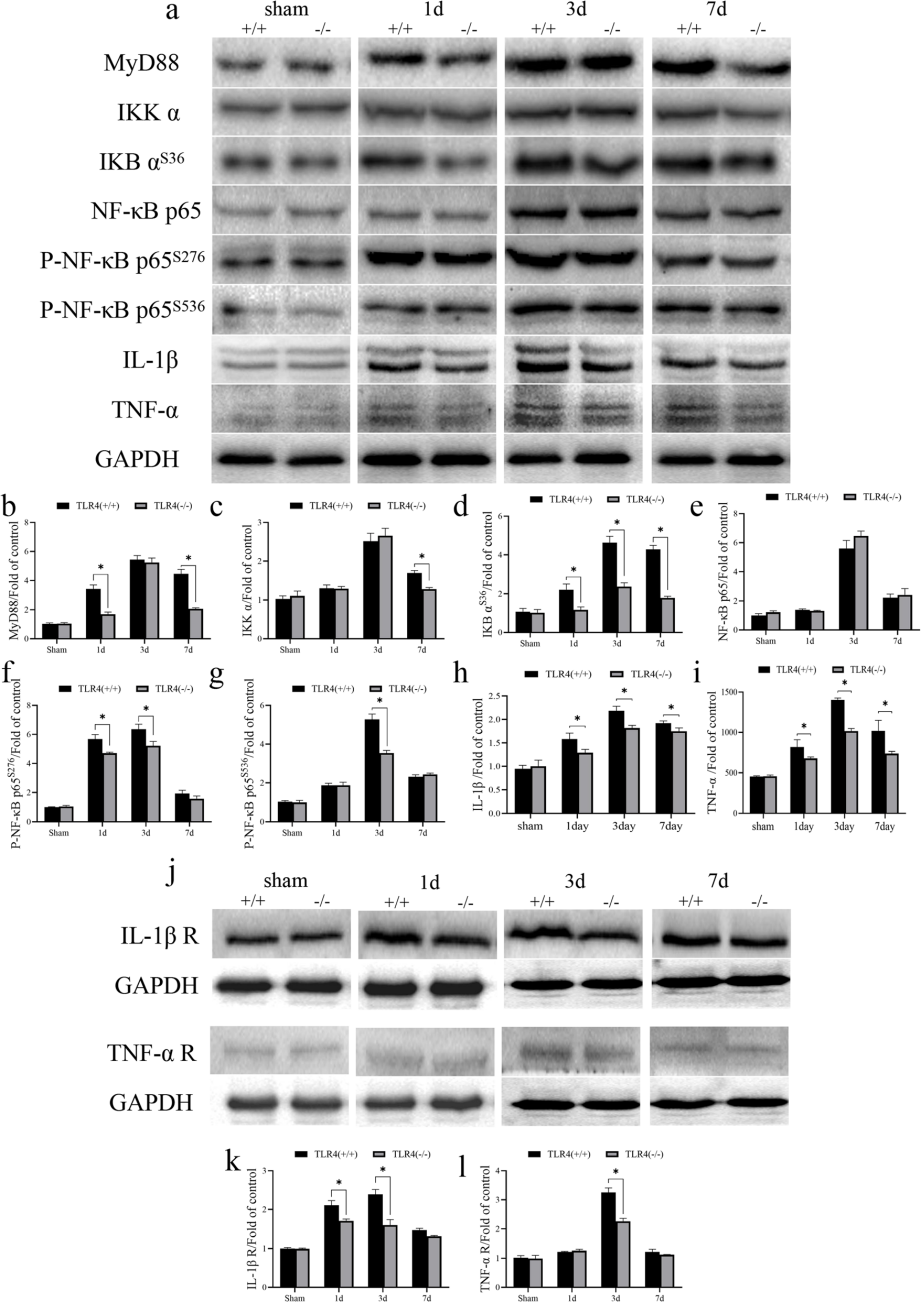
Expression of inflammatory cytokines and their receptors in brain tissue of TLR4 knockout mice and wild-type mice after inducing ICH. **a** The expressions of MyD88, IKK α, IKB α^S36^, NF-κB, IL-1β, and TNF-α in brain tissue of TLR4 knockout mice and wild-type mice on day 1, 3, and 7 after inducing ICH. **b** Quantification of MyD88. **c** Quantification of IKK α. **d** Quantification of IKB α^S36^. **e** Quantification of NF-κB p65. **f** Quantification of P-NF-κB p65^S276^. **g** Quantification of P-NF-κB p65^S536^. **h** Quantification of IL-1β. **i** Quantification of TNF-α. **j** The expression of IL-1β receptor and TNF-α receptor in brain tissue of TLR4 knockout mice and wild-type mice was detected on day 1, 3, and 7 after inducing ICH. **k** Quantification of IL-1β R. **l** Quantification of TNF-α R. GAPDH is used as a housekeeping protein to prove the equal loading in each lane and normalize densitometric values of the other protein analyzed. Each experiment was repeated three times. *P < 0.05 represents a statistically significant difference between the two groups

Triggering the TLR pathway often leads to the activation of NF-κB and subsequent regulation of immune and inflammatory genes [9]. MyD88, IKK α, IKB α^S36^, NF-κB p65, NF-κB p65^S276^, and NF-κB p65^S536^ are key regulators in the TLR4/NFκB signaling pathway. Therefore, we wanted to explore whether TLR4 regulates apoptosis through inflammatory mechanisms. We examined the expression of MyD88, IKK α, IKB α^S36^, NF-κB p65, NF-κB p65^S276^, and NF-κB p65^S536^ inflammatory factors in the tissue surrounding the bleeding site of the mouse at time points corresponding to those of the previous experiment and found that regulatory proteins in the TLR4/NF κB pathway and the inflammatory factors TNF-α and IL-1β increased significantly after ICH induction and peaked on the third day (Fig. 4a). In addition, the expression of MyD88 was different in the two groups on the first and seventh days after surgery (Fig. 4b). The expression of IKK α was different in the two groups on the seventh day after surgery (Fig. 4c). The expressions of IKB α^S36^, IL-1β, and TNF-α were different in the two groups on the first, third, and seventh days after surgery (Fig. 4d,h,i). Although the expression of NF-κB p65 was upregulated after ICH induction, we did not find statistical differences in the expression in both groups (Fig. 4e). Phosphorylation at different sites of NFκB p65 leads to its activation. The expressions of NF-κB p65^S276^ and NF-κB p65^S536^ were different in the two groups on both first and third days and only the third day, respectively, after surgery (Fig. 4f and g, respectively). In addition, we examined the expression of IL-1β and TNFα receptors and found a significant up-regulation of expression after ICH induction with TLR4 (−/−) mice showing lower expression compared to wild-type mice (Fig. 4j). After quantifying the expression of IL-1β and TNF-α receptors, we found that IL-1β receptor expression was different in the two groups on postoperative days 1 and 3 (Fig. 4k). On the postoperative day 3, TNFα receptor expression was different in the two groups (Fig. 4l). Therefore, we hypothesize that TLR4-mediated apoptosis may be associated with inflammation.

### Inflammatory factor antagonists inhibit TLR4-induced apoptosis

**Fig. 5:**
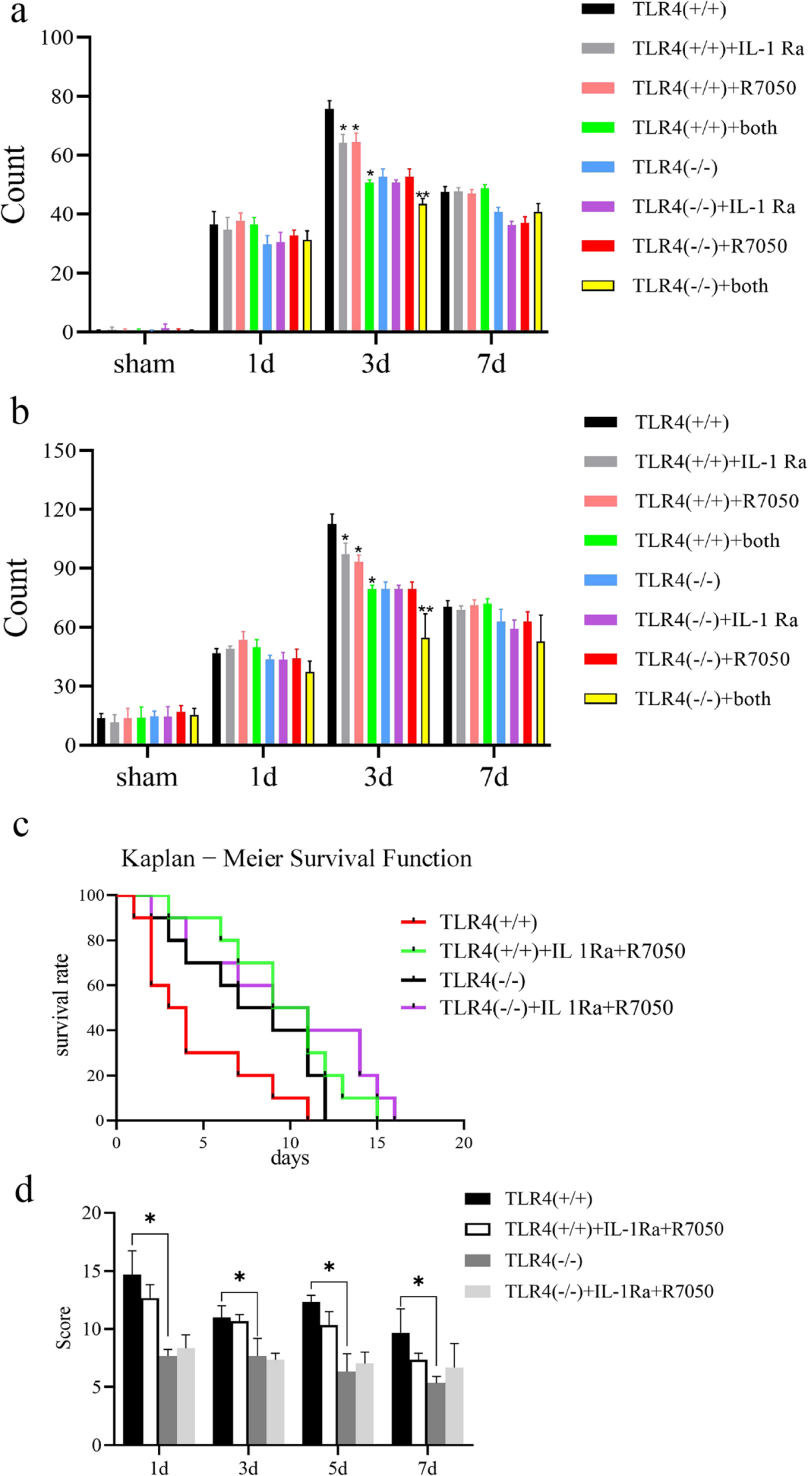
Brain tissue apoptosis after injection of inflammatory factor antagonist. **a** TUNEL and **b** Hoechst staining of brain tissue of ICH-induced mice on day 1, 3, and 7 after injecting IL-1Ra, or R7050, or both. The number of apoptotic cells in four different fields counted under a microscope. *P < 0.05 vs. TLR4(+/+) group 3 days after induction of ICH. **P < 0.05 vs TLR4(−/−) group 3 days after induction of ICH. **c** Kaplan-Meier survival analysis on ten mice for each group. **d** Behavioral Longa scores of ten mice for each group. *P < 0.05 represents a statistically significant difference between the two groups

Previous experimental results have fully demonstrated that TLR4 is a key molecule in inducing brain apoptosis after ICH induction. To further verify whether TLR4 exerts a pro-apoptotic effect through the inflammatory pathway, we injected 3 mg/kg IL-1RA (MedChemExpress; China) or R-7050 (MedChemExpress; China) 30 min after induction of ICH. We performed TUNEL (Fig. 5a) and Hoechst (Fig. 5b) staining techniques after sacrificing mice on days 1, 3, and 7 after inducing ICH. On days 1 and 7 after injection of inflammatory factor antagonists, there was no significant difference in the number of apoptotic cells in the two groups. On the third postoperative day, the number of apoptotic cells was significantly reduced in wild-type mice injected with IL-1Ra or R7050 compared with the control group. Simultaneous injection of IL-1Ra and R7050 showed a more pronounced effect of inhibiting apoptosis. In TLR4(−/−) mice, inhibition of apoptosis was observed only by simultaneous injection of IL-1Ra and R7050. Although statistically significant, the inhibitory effect of apoptosis in TLR4(−/−) mice was not as pronounced as in wild-type mice. Although the principles of TUNEL and Hoechst staining are different, both staining results show that IL-1RA or R-7050 can effectively inhibit cell apoptosis and the inhibitory effect is weakened after knocking out the *TLR4* gene.

We also performed survival analysis on each group of mice and found that the survival rate of TLR4(+/+) mice was significantly lower than that of TLR4(−/−) mice, which is consistent with our previous conclusions (Fig. 1f). The survival rate of TLR4(+/+) mice injected with IL 1Ra and R7050 was significantly increased (Mantel-Cox test, Chi square: 12.17, *P* = 0.0068). Compared with the TLR4(−/−) group, the long-term survival rate of TLR4(−/−) mice injected with IL 1Ra and R7050 was improved, but there was no significant difference between the recent survival rates (Fig. 4c). Unfortunately, in the behavioral scoring experiments, we did not observe changes in behavioral symptoms after injection of antagonists in TLR4 (−/−) and TLR4 (+/+) mice (Fig. 4d).

## Discussion

Inflammation and apoptosis induced after ICH induction are important factors affecting the quality of life and prognosis of patients. The relationship between inflammation and apoptosis is very complicated. Inflammation can cause apoptosis and apoptotic cells can also cause inflammation in a microenvironment.

Our study found that TLR4 increases the expressions of IL-1β and TNF-α and their receptors after ICH induction. In ICH-induced mice lacking TLR4, the expression of inflammatory factors and their receptors was attenuated. In addition, wild-type mice had significantly more neuronal apoptosis after ICH induction than the TLR4 knockout mice. Apoptosis in wild-type mice is inhibited by IL-1β and TNF-α antagonists IL-1RA and R-7050. In the TLR4 knockout mice, the single injection of one antagonist had no significant effect and only the simultaneous injection of IL-1RA and R-7050 showed a slight inhibitory effect indicating that TLR4 can promote apoptosis after ICH induction and the underlying mechanism may be related to its pro-inflammatory effect. Clinically, the current treatment of cerebral hemorrhage mainly uses hemostatic drugs such as hemocoagulase and drugs that reduce intracranial pressure dehydration treatment such as mannitol. However, there is no effective treatment for secondary damage such as inflammation on brain tissue. Our study found that in TLR4 (+/+) mice, in situ injection of IL-1Ra or R7050 showed good therapeutic effects, and the combination therapy was more effective. In addition, compared with TLR4 (+/+) mice, although the efficacy was not as obvious in TLR4(−/−) mice, the combination of the two also showed therapeutic effects. Despite the limited therapeutic effect, patients can be clinically given fewer complications and a better prognosis.

The relationship between TLR4 and apoptosis has been studied in many diseases, especially in the field of oncology. Although many researchers have explored the relationship between TLR4 and apoptosis in ICH, unfortunately, almost all studies have introduced a third variable. Therefore, we cannot speculate whether the relationship between TLR4 and apoptosis is due to the introduced variables. After making the ICH model, we injected IL-1Ra and R7050 antagonists into TLR4(+/+) mice. Although we did not observe significant improvement in behavioral behavior, we found that the survival rate of mice and apoptosis of brain tissue were significantly improved after injection. This apparent improvement in survival did not exist in TLR4(−/−) mice (Fig. 5c, d). IL-1Ra and R7050 are inflammatory factor antagonists and although there are many factors that cause inflammatory factor release in ICH, these two antagonists exhibit an effective therapeutic effect in the presence of TLR4. Therefore, we believe that the inflammatory response to brain tissue damage caused by ICH is mainly mediated by TLR4. Also, the inflammatory factor antagonists IL-1Ra and R7050 may play a therapeutic role through TLR4, which provides sufficient evidence for clinical treatment of cerebral hemorrhage. Otherwise, many studies have explored the therapeutic effects of drugs on ICH, but most of the modes of administration are pre-administration by intraperitoneal injection. Clinically, ICH is often caused by factors such as high blood pressure or craniocerebral trauma. It is often unpredictable, thus, pre-administration is not in line with clinical practice. Clinically, mild ICH is treated with conservative observation and severe ICH is surgically treated. The drug needs to pass through the blood-brain barrier through intraperitoneal injection, which has a great interference with the efficacy of the drug. In situ administration of drugs during surgery seems to be more effective and clinically convenient to treat severe ICH. However, such a mode of administration also has shortcomings. In view of the importance of brain tissue, the toxic and side effects of drugs on central nervous cells should be considered, which is a shortcoming of our research.

TLR4 recognizes and initiates an LPS-triggered immune response to gram-negative bacteria [11]. It also triggers activation of NFκB, IRF-3, and MAPK pathways, causing inflammatory cytokine synthesis [13]. The nuclear factor κB (NF-κB)/Rel family of transcription factors play an important role in inflammation and immune response [21, 22]. There are five family members in mammals: p65 (RelA), c-Rel, RelB, NF-κB1 (p105/p50), and NF-κB2 (p100/p52). Both p105 and p100 can be proteolytically treated with proteasome to produce p50 and p52, respectively. The Rel protein binds to p50 and p52 to form a dimer, which in turn binds to DNA and regulates transcriptional activity. NF-κB is blocked in the cytoplasm by IκB inhibitory proteins in unstimulated cells [23–25]. NF-κB activator induces phosphorylation of IκB protein and rapidly degrades its target through the ubiquitin–proteasome pathway, releasing NF-κB into the nucleus and regulating gene expression [26]. NIK and IKKα (IKK1) regulate phosphorylation and processing of NF-κB2 (p100) to produce p52, which is then translocated into the nucleus [27, 28]. Phosphorylation at different sites of NFκB p65 leads to its activation. Although we found no significant difference in the expression of NF-κB p65 between TLR4(−/−) and wild-type mice at various time points after surgery, the expression of NF-κB p65^S276^ was different in the two groups on the first and third day after surgery (Fig. 4f). The expression of NF-κB p65^S536^ was different in the two groups on the third day after surgery (Fig. 4g). Most of the research on phosphorylation of RelA/p65 in recent years has focused on the Ser276 and Ser536 sites. In addition, there are currently 11 phosphorylation sites for RelA/p65. Four sites Ser205, Thr254, Ser276, and Ser281 are located in RHD, and seven sites Thr435, Ser468, Thr505, Ser529, Ser535, Ser536, and Ser547 are located in the TAD region. Ser316 and Ser311 are located at the junction of the RHD end. In addition, TAD has traditionally been divided into two distinct transactivation domains, TA1 and TA2, which have been shown to be transcriptional activation regions of RelA/p65. TA1 is located at the C-terminal portion of TAD (residues 521–551) and TA2 is located in the first 90 amino acids (residues 428–521) [29]. The highly conserved Ser276 residue can be stimulated by various inducers such as LPS and TNF and phosphorylated under the action of PKAc, thereby coordinating phosphorylation of IkB complex and degradation of IKBα [30]. Ser536 can be phosphorylated by IKKα, IKKβ, IKKε, NAK, and RSK1; enhances transcriptional activity by increasing binding to CBP/p300 and K310 acetylation; and has an effect on the stability of nuclear translocation [30]. Although we found that the overall content of NFκB was not statistically different between the two groups, it showed significant differences in Ser276 and Ser536 phosphorylation. This is also in line with our hypothesis that antagonists exert an anti-inflammatory effect through the TLR4/NFκB pathway. In addition, as for the phosphorylation of NFκB p65 subunit, there are indeed difference between the TLR4 (+/+) and TLR4 (−/−) mice which suggest the possible contribution for TLR4 in p65 modification (Fig. 4a), but there existed the remarkable difference between the SHAM and ICH groups no matter in TLR4(+/+) and TLR4(−/−)mice, which hints that the TLR4 is not the whole story in NF-kB-mediated neuroinflammation induced by ICH. Other inflammatory mechanisms associated with NF-kB pathway induced by ICH remain to be studied.

Current research suggests that there are two main pathways for causing apoptosis: the extracellular death receptor pathway and the intracellular mitochondrial pathway [31]. The mechanism of action of NF-κB in apoptosis is complex. It is involved in the transcriptional regulation of various apoptosis-related genes and has a two-way effect of inhibiting and promoting apoptosis [32, 33]. NF-κB anti-apoptosis involves multiple signaling pathways, but its main mode is achieved by inducing or upregulating the expression of anti-apoptotic genes. These gene regulatory sites have binding sites for NF-κB and their expression products act through a death-receptor pathway or a mitochondrial pathway that inhibits apoptosis. So far, it has been found that the anti-apoptotic pathway after NF-κB activation involves at least zinc finger protein A20 [34], superoxide dismutase, BCL-2 homologue, and TNFR-related factor [35].

However, NF-κB also plays a role in promoting apoptosis [33], which may be due to NF-κB-stimulated apoptosis-inducing genes such as c-IAP, P53, c-Myc, and Fas. At the same time, NF-κB can directly induce IL-1, TNF-α, and other inflammatory mediators and enzymes that promote apoptosis. Studies have shown that NF-κB can enhance P53-induced apoptosis in many cell types [36]. Tumor suppressor ARF regulates NF-κB activity through ATR/Chk1 activation, thereby inhibiting Bcl-xL expression, and ultimately enhances TNF-induced cell apoptosis [37]. To put it another way, considering that NF-κB has multi-directional transcriptional regulation as a nuclear factor, the contradictory role of NF-κB in apoptosis is also understandable.

Inflammation can cause apoptosis and vice versa. We found that TLR4 induces inflammation after ICH induction and an increase in apoptosis. The effect of inflammation on apoptosis was verified by antagonists. However, in view of the regulatory complexity of downstream molecules NF-κB, the specific mechanism of TLR4 in inflammation and apoptosis after ICH induction remains to be further explored.

## Conclusion

In conclusion, we found that TLR4 mediates apoptosis by inducing an inflammatory response after ICH induction. In patients with clinical ICH, targeting TLR4 and using inflammatory factor antagonists may attenuate the inflammatory response, thereby attenuating apoptosis and improving prognosis.

## Data Availability

Not applicable.

## Abbreviations

ICH: Intracerebral hemorrhage
LPS: Lipopolysaccharide
NF-κB: Nuclear factor κB
TLR4: Toll-like receptor 4
